# Chronic HIV Infection Associated With Motor Neuron Disease and Parkinsonism: A Case Report and Narrative Review

**DOI:** 10.7759/cureus.89530

**Published:** 2025-08-07

**Authors:** Pedro V Sabbadini, Marco A Orsini, João Carlos Amorelli Figueira, Omar H Ashmawi, Amanda Amorelli Fernandes, Joao V Costa, Thiago De Mello Tavares, Marcos De Freitas, Acary S Oliveira, Billy McBenedict

**Affiliations:** 1 Neurology, Fluminense Federal University, Niterói, BRA; 2 Neurology, Federal University of Rio de Janeiro, Rio de Janeiro, BRA; 3 Neurology, UNIG - Iguaço University, Rio de Janeiro, BRA; 4 General Surgery, Petrópolis School of Medicine, Petrópolis, BRA; 5 Family Medicine, Federal University of Santa Catarina, Florianópolis, BRA; 6 Neurology, Federal University of São Paulo, São Paulo, BRA; 7 Neurosurgery, Fluminense Federal University, Niterói, BRA

**Keywords:** chronic hiv, extrapyramidal syndromes, motor neuron disease (mnd), neurodegeneration, parkinsonism, pyramidal syndromes

## Abstract

Motor neuron disease and parkinsonism syndromes associated with chronic HIV infection are extremely rare, with only a few cases reported in major medical databases. We report a case from Brazil involving a patient with a chronic, well-controlled HIV infection for approximately 15 years, demonstrating a good response to highly active antiretroviral therapy. Despite the absence of viral replication, the patient developed atypical clinical and topographical features consistent with both pyramidal and extrapyramidal syndromes. This raises the question of whether such findings could be linked to chronic HIV infection, occurring simultaneously even in the absence of active viral replication, particularly given that other potential diagnoses were excluded.

## Introduction

Approximately 39.9 million people worldwide are currently living with HIV [1,2], and 29 million have access to antiretroviral therapy (ART) [3], which helps control viral load while maintaining CD4 T cell counts. Neurological complications are common in HIV, occurring in up to 70% of autopsy cases. These complications may result from the direct effects of the virus, immune-mediated responses, opportunistic infections, or side effects of ART [3]. Several studies have established a direct association between the presence of HIV antigens in the brain and neuronal degeneration [4]. This degeneration is driven by by-products of HIV infection - such as gp120, Nef, and Tat proteins - as well as the increased production of free radicals, cytokines, bioactive lipid mediators, excitotoxic amino acids, and disruption in neuroprotective mechanisms [5,6].

If left untreated, HIV infection progresses through three stages: primary infection, clinically latent disease, and AIDS. There is typically a latency period of 8-12 years between primary infection and the development of AIDS. However, the advent of newer antiretrovirals has significantly improved survival rates, leading to increased longevity and aging among people living with HIV [2]. This extended lifespan raises the likelihood of overlap between age-related conditions and those potentially triggered by chronic HIV infection, such as motor neuron disease (MND), which remains rare in this population [7]. Additionally, the long duration of HIV infection in this patient brings attention to phenomena such as “blips,” which are transient elevations in viral load (typically 50-200 copies/mL). Even at these low levels, blips may cause cumulative damage to the nervous system over time [8].

The aim of this report is to highlight a case in which HIV may have been the primary trigger for parkinsonism and MND, especially given that all other differential diagnoses were thoroughly excluded.

## Case presentation

The patient was a 63-year-old woman with a medical history limited to mild systemic arterial hypertension and hepatic steatosis. She had been living with chronic HIV infection for approximately 15 years, with sustained viral suppression and preserved immune function, as evidenced by CD4+ lymphocyte counts consistently above 500 cells/mm³. There was no family history of neurodegenerative diseases or movement disorders. One sister had breast cancer (in remission), her brother was healthy, and both parents had died of cardiovascular disease.

Neurological symptoms began insidiously in early 2023, approximately 14 years after her HIV diagnosis. According to family members, the initial signs included generalized slowness and intermittent resting tremor. Neurological care was initially sought through the private healthcare system, while HIV follow-up continued through the public health system (Sistema Único de Saúde, SUS), in accordance with national protocols.

Over the following months, her symptoms progressively worsened. By May 2024, she presented with a combination of pyramidal and extrapyramidal features, including generalized paresis, spasticity, hyperreflexia, clonus, positive Babinski and Hoffman signs, bradykinesia, hypomimia, postural instability, freezing episodes, resting tremor, truncal flexion, and loss of protective reflexes. Sensory examination revealed tactile and thermal hypoesthesia, diminished proprioception, and hypopalesthesia. She also reported persistent burning dysesthesia in both lower limbs.

Treatment with dopaminergic agents, including levodopa and pramipexole, was initiated but failed to produce any significant clinical improvement. The lack of response raised suspicion of an atypical parkinsonian syndrome. This prompted further investigation by the infectious disease and general medicine teams, given her longstanding HIV infection.

A systematic diagnostic workup was conducted to exclude secondary causes of parkinsonism. Neuroimaging (brain MRI) revealed no evidence of ischemic lesions, demyelination, neoplasms, basal ganglia calcification, or structural abnormalities. There were no findings suggestive of progressive supranuclear palsy (PSP), multiple system atrophy (MSA), or other atypical parkinsonian syndromes. Cranial nerves and cognitive function were preserved. Electroneuromyography (ENMG) demonstrated normal sensory and motor nerve conduction (Table 1, Table 2).

**Table 1:** Sensory nerve conduction studies Sensory nerve conduction studies showed normal amplitude and latency values in the radial, median, ulnar, superficial fibular, and sural nerves. Sensitization tests for carpal tunnel syndrome, including comparison of sensory latencies between the first and fourth digits, were negative bilaterally.

Nerve/site	Recording site	Onset latency (ms)	Peak latency (ms)	NP amplitude (µV)	PP amplitude (µV)	Distance (mm)	Peak difference (ms)	Velocity (m/s)
R. median, radial - thumb comparison	Radial wrist	2.05	2.85	7.7	7	100		49
R. median, radial - thumb comparison	Thumb							
R. median, radial - thumb comparison	Median wrist	2.5	2.95	20.5	28.8	100		49
R. median, radial - thumb comparison	Thumb						0.1	
L. median, radial - thumb comparison	Radial wrist	2.35	3.1	4.7	6.2	100		43
L. median, radial - thumb comparison	Thumb							
L. median, radial - thumb comparison	Median wrist	2.45	3.25	18.8	21.8	100		41
L. Median, Radial - Thumb Comparison	Thumb						0.15	
R. median - dig III (antidromic)	Wrist	2.75	3.55	28.3	45.2	140		51
R. median - dig III (antidromic)	Digit III							
L. median - dig III (antidromic)	Wrist	2.7	3.45	26.8	41.7	140		52
L. median - dig III (antidromic)	Digit III							
L. median - dig III (antidromic)	Palm	1.3	1.95	31.2	46.5	70		54
L. median - dig III (antidromic)	Digit III							
R. ulnar - dig V (antidromic)	Wrist	2.7	3.5	27.5	33.5	140		52
R. ulnar - dig V (antidromic)	Digit V							
L. ulnar - dig V (antidromic)	Wrist	2.65	3.4	25.7	37.7	140		53
L. ulnar - dig V (antidromic)	Digit V							
L. superficial peroneal (antidromic)	Lat Leg	2.04	2.7	14.7	16.3	120		59
L. superficial peroneal (antidromic)	Ankle							
R. superficial peroneal (antidromic)	Lat leg	2.3	2.94	19.2	13.3	120		52
R. superficial peroneal (antidromic)	Ankle							
R. sural (antidromic)	Calf	1.95	2.8	18.7	12.7	140		72
R. sural (antidromic)	Ankle							
L. sural (antidromic)	Calf	2.3	3	17.3	24.7	140		61
L. sural (antidromic)	Ankle							

**Table 2:** Motor nerve conduction studies Motor nerve conduction studies showed normal amplitude, latency, and conduction velocity in the median, ulnar, tibial, and fibular nerves. F-wave studies demonstrated normal minimum latency values in the median and tibial nerves. The H-reflex (S1 root) was within normal limits bilaterally. EDB, extensor digitorum brevis

Nerve/site	Recording site	Latency (ms)	Amplitude (mV)	Rel amp %	Dur. (ms)	Dur. %	Distance (mm)	Velocity (m/s)	Area (mVms)	Area %
R. peroneal - EDB	Ankle	2.86	4.03	100	5.2	100	80		13.6	100
R. peroneal - EDB	B. fib head	8.42	3.09	90.5	5.4	103	280	50	12.6	93.2
L. peroneal - EDB	Ankle	3.02	3.3	100	5.7	100	80		10.5	100
L. peroneal - EDB	B. FIB HEAD	8.8	3.2	96.6	6.7	116	280	48	10.6	100
L. tibial - AH	Ankle	3.26	15.2	100	5.2	100	80		29.2	100

**Figure 1:**
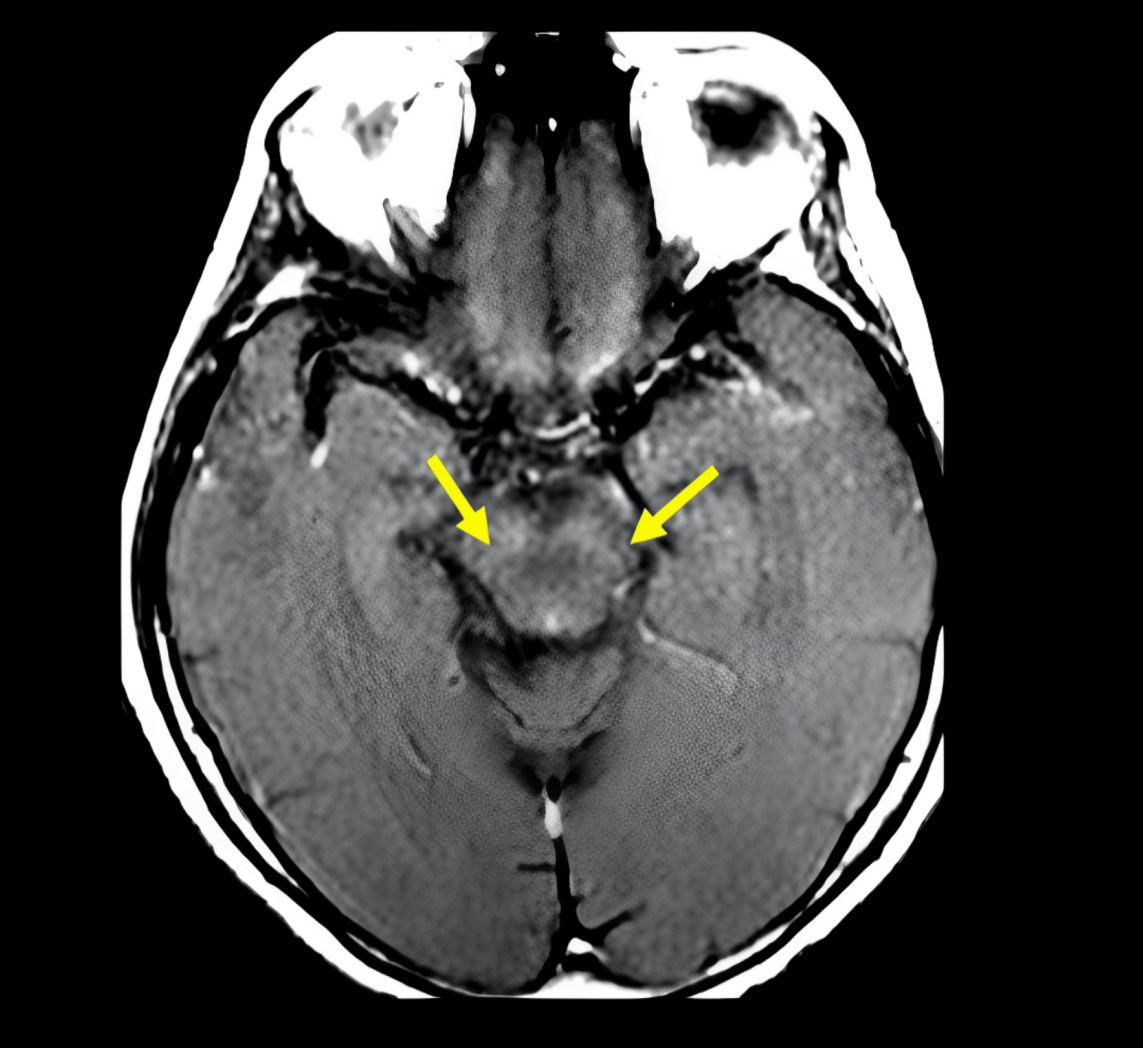
MRI showing a mesencephalic lesion and thinning of the substantia nigra

**Figure 2:**
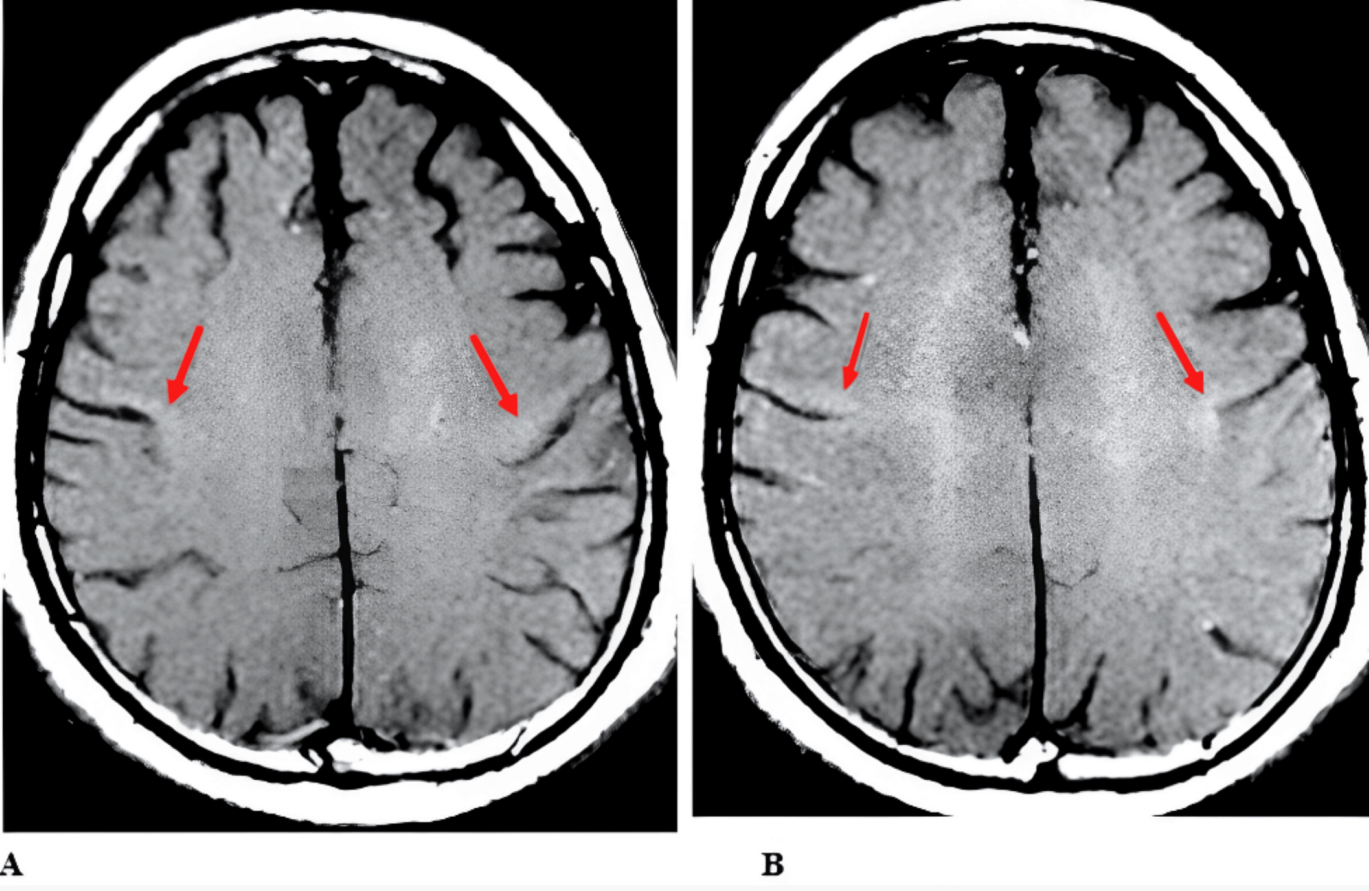
T1-weighted sequence with magnetization transfer technique showing a subtle hypersignal along the pyramidal pathway (arrows in A and B)

**Figure 3:**
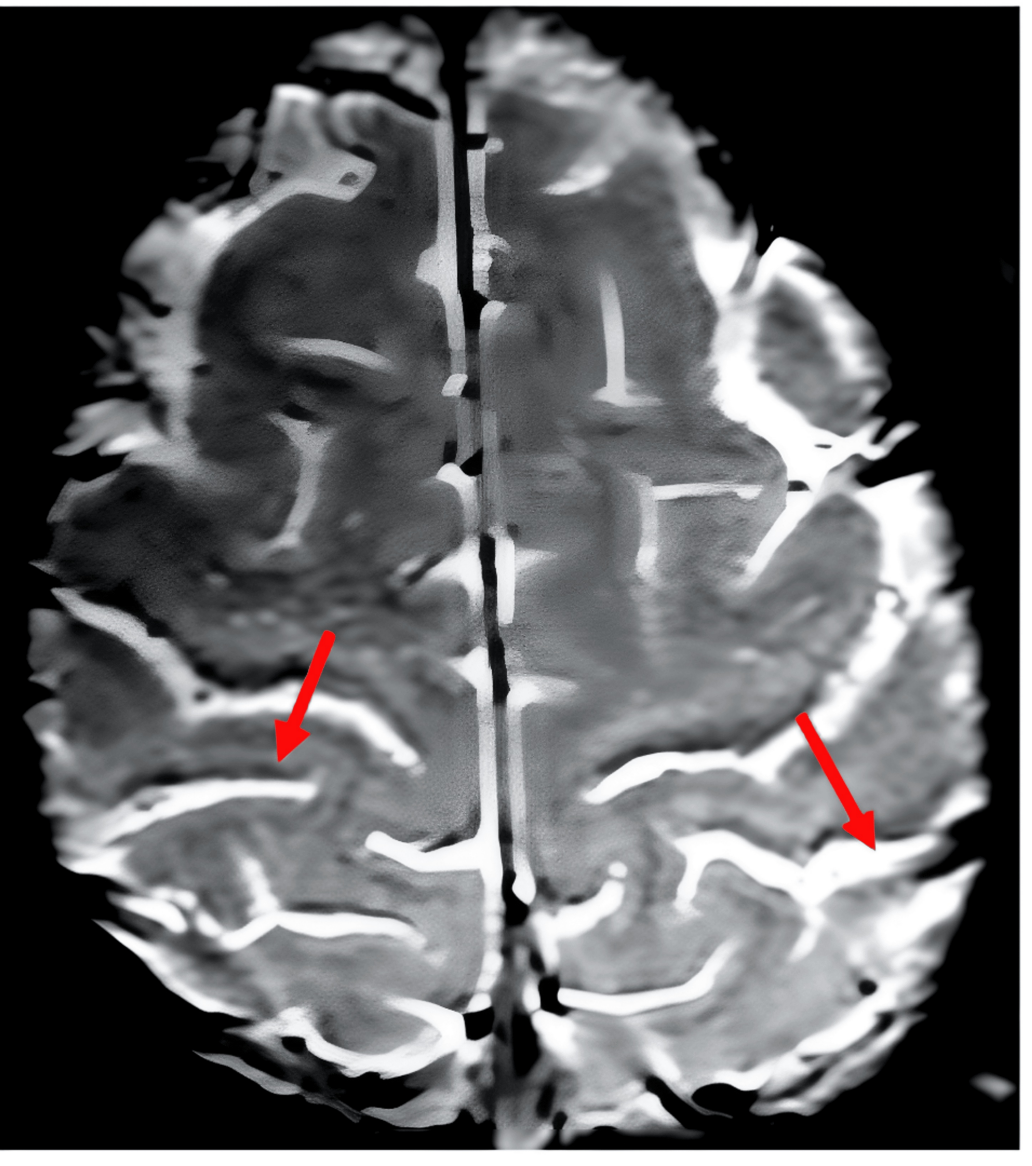
Magnetic susceptibility imaging shows marked hypointensity in the topography of the precentral gyrus cortex, a finding commonly observed in upper MNDs MND, motor neuron disease

MRI revealed signal alterations in the mesencephalon and basal ganglia (Figure 1), as well as a discrete hypersignal in the T1 sequence using the magnetization transfer technique along the pyramidal pathway (Figure 2). Additionally, the imaging showed topographic features indicative of upper MND (Figure 3). It is worth noting that no neurophysiological signs of lower motor neuron involvement were identified on ENMG, such as neurogenic patterns, positive sharp waves, high-amplitude and long-duration potentials, fasciculations, or fibrillations.

Serologic and CSF studies were non-reactive for cytomegalovirus, herpes simplex virus, syphilis, toxoplasmosis, varicella-zoster virus, *Cryptococcus neoformans*, other fungi, and bacteria. CSF glucose measured 61 mg/dL, and no abnormalities were found in paraneoplastic, neoplastic, or autoimmune biomarkers. In this context, the coexistence of pyramidal and extrapyramidal signs in a patient with well-controlled HIV infection and no other plausible etiologies supported the hypothesis of a chronic HIV-associated neurodegenerative process. Although her age did not exclude idiopathic Parkinson’s disease, the constellation of findings, especially the unresponsiveness to dopaminergic therapy and absence of structural lesions, suggested an alternative mechanism. It was therefore conceivable that intermittent viral reactivation (“blips”) or chronic exposure to residual neuroinflammatory mediators contributed to progressive neuronal injury. While this remained a hypothesis, it aligned with the observed clinical pattern and the exclusion of other causes.

## Discussion

The case presented suggests a possible association between chronic HIV infection and a progressive motor syndrome characterized by both pyramidal and extrapyramidal features, consistent with parkinsonism, even in the absence of detectable viral load. Although a definitive causal link cannot be established, the exclusion of other etiologies - vascular, autoimmune, neoplastic, or toxic - supports the plausibility of HIV-related neurodegeneration as a contributing factor.

Quevedo-Ramirez et al. [9] described a case involving a 38-year-old man from Lima, Peru, who was diagnosed with HIV (CD4 baseline: 220 cells/mm³; viral load: 67,019 copies/mL) and initiated on combined ART (cART). Despite the low viral load, the patient’s neurological condition progressively worsened over the course of a year, ultimately resulting in death.

One possible diagnosis in such scenarios is immune reconstitution inflammatory syndrome (IRIS), which is characterized by TCD8+ lymphocytic infiltration (although this is not a formal diagnostic criterion) [9]. It is hypothesized that IRIS, in the absence of opportunistic infections, may trigger an autoimmune response affecting both the central and peripheral nervous systems, leading to long-term neurological damage. This immune response is driven by TCD8+ cell infiltration following the rapid recovery of TCD4+ lymphocytes after initiation of highly active ART (HAART) [9]. The paradoxical deterioration of health following cART initiation - seen in approximately 13% of HIV patients - supports the potential role of IRIS in the worsening of neurological conditions [10]. While HAART has reduced neuropathies caused by coinfections, cases of encephalitis linked to immune activation have increased, as revealed by autopsy analyses, emphasizing the importance of understanding these underlying mechanisms [10].

Cytokines like IL-16, which increase immune reactivity to neural tissue, and neurotoxic substances released by activated microglia - including excitatory amino acids, quinolinate, cysteine, and a partially characterized compound named Ntox - have been directly implicated in neural apoptosis, a process known as excitotoxicity [11]. This harmful process involves excessive Ca²⁺ influx and neuronal and glial apoptosis, mediated by the HIV-1 Tat protein, which disrupts intracellular calcium homeostasis - a mechanism also involved in the pathophysiology of MNDs such as ALS [12]. HIV-1 proteins, including Tat and gp120, have well-documented neurotoxic effects. The presence of antibodies against these proteins correlates with greater neurological impairment, and their production persists even when HIV RNA levels are undetectable in plasma [12].

Disruption of calcium homeostasis contributes to mitochondrial dysfunction in neurons and glial cells, which are highly reliant on mitochondrial activity. HIV-related proteins like Tat and gp120 can impair mitochondrial function by promoting fragmentation and reducing membrane potentials [12]. Furthermore, overstimulation of glutamate receptors leads to free radical production (e.g., nitric oxide and superoxide anion), which exacerbates neuronal apoptosis [4,13].

Amyotrophic lateral sclerosis (ALS), the most common MND in adults, is significantly more prevalent in individuals with HIV, approximately 3.5 cases per 1,000 patients, nearly 100 times the general population rate [14]. ALS also exhibits the most favorable therapeutic response among MNDs, making it a key condition for understanding HIV-associated motor syndromes. Most HIV-related ALS cases occur in the context of advanced immunodeficiency and high viral loads in both plasma and CSF, although cases without detectable viral load have been reported. These syndromes are believed to result from increased viral replication in the CNS, and clinical improvement is typically observed when potent antiretroviral regimens reduce HIV RNA levels in plasma and CSF [15].

However, not all patients with HIV-related ALS or MND benefit from HAART. Calza et al. [15] reported a case of ALS-like syndrome in an HIV-infected patient who improved after starting HAART (CD4: 721 cells/mm³; HIV viral load <50 copies/mL). Nonetheless, 15 months later, the patient experienced worsening neurological symptoms, including severe dysarthria, dysphagia, and tetraparesis, despite a stable immune profile and undetectable viral load, ultimately leading to respiratory failure and death.

Even with HAART-induced viral suppression and immune recovery, approximately 50% of patients continue to experience HIV-associated neurocognitive disorders [16]. In the context of our case - with upper MND and thinning of the substantia nigra - it is pertinent to explore the relationship between parkinsonism and HIV. Although motor syndromes, including Parkinson’s disease, are rare in this population, they affect around 5% of HIV-infected individuals. Moreover, extrapyramidal symptoms resembling parkinsonism have been reported in treated populations [16]. Our patient, aged 63 and undergoing treatment for 15 years, demonstrated signs such as substantia nigra thinning and upper motor neuron involvement, raising the possibility of progression to atypical parkinsonian syndromes like MSA.

A key question is the extent to which neurodegeneration is driven by aging versus chronic inflammation induced by HIV. The observed regression of parkinsonian symptoms following HAART and the normalization of TCD4 counts [16] support a connection between HIV-related inflammation and neurodegeneration. Contributing factors such as oxidative stress, telomere shortening, and mitochondrial dysfunction [17] may further accelerate neuronal damage. The basal ganglia, particularly the substantia nigra, is highly susceptible to HIV-related inflammation, with up to 25% neuron loss documented during AIDS [18]. Postmortem studies of HIV patients on HAART have shown significant dopamine depletion in the substantia nigra, caudate, putamen, and globus pallidus - regions implicated in both Parkinson’s disease and the parkinsonism observed in this case [16].

Case studies and reviews support the hypothesis that HIV may mimic ALS or primary lateral sclerosis (PLS) phenotypes through neuroinflammation and degeneration [19]. Persistent immune activation and the release of inflammatory mediators may cause upper motor neuron degeneration characteristic of PLS [19]. Our case likely aligns with this presentation.

PLS is a slowly progressive neurodegenerative disease affecting the central motor system, leading to muscle stiffness, mobility loss, and corticobulbar dysfunction. UMN-predominant ALS shows a similarly slow progression, with survival extending beyond a decade after symptom onset [20].

Another neurodegenerative disease with potential relevance to HIV is MSA. This progressive and degenerative proteinopathy results from the accumulation of insoluble fibrillary alpha-synuclein (aSyn) and is characterized by parkinsonism combined with dysfunction of the cerebellum and autonomic nervous system. It affects both sexes equally and typically presents in the sixth decade of life. MSA is subdivided into two main clinical variants: the parkinsonian type (MSA-P) and the cerebellar type (MSA-C) [17,18]. For the purpose of this discussion, MSA-P is of particular interest, as the patient in this case exhibited clinical features suggestive of this subtype, including parkinsonism with bradykinesia, spasticity, and the presence of Babinski’s sign [17].

In MSA, alpha-synuclein accumulates in neurons and oligodendrocytes, triggering CNS inflammation, which plays a key role in disease pathogenesis. One of the primary mechanisms involves microglial activation in response to the aSyn aggregates. These activated microglia can assume either a pro-inflammatory or anti-inflammatory phenotype, releasing corresponding cytokines detectable in brain parenchyma, blood, and CSF. In the pro-inflammatory state, IL-2, IL-13, and T cells (CD3+, CD4+, CD8+) are often found in the CNS.

Simultaneously, dysfunction in oligodendrocytes - cells responsible for myelin production and neuronal support - impairs their ability to maintain homeostasis. Under metabolic stress, these cells shift from producing myelin to prioritizing their own survival [21]. Moreover, the upregulation of interferon-stimulated genes and continued inflammation, exacerbated by aSyn accumulation, lead to enhanced transcription of inflammatory mediators. However, it is important to note that blood and CSF levels of these mediators are not always altered in patients with MSA [18].

Astrocytic inflammation in the CNS is also a feature of MSA, with increased mitochondrial production and respiratory activity. In animal models, age-related metabolic changes in astrocytes - such as elevated mitochondrial aerobic activity - may impair their ability to supply neurons with glucose, exerting a neurotoxic effect. Since individuals living with HIV are in a state of chronic CNS inflammation, this mechanism could partly explain the neurological deterioration observed in this case [12].

HIV itself is known to cause both neuronal and oligodendrocyte injury through the action of viral proteins and secondary inflammatory responses, leading to calcium influx and cellular dysfunction - parallels that MSA shares, though caused by different primary factors (i.e., aSyn deposition) [12,18].

To meet the diagnostic criteria for MSA (clinically established, probable, or possible prodromal), the presence of autonomic dysfunction or cerebellar syndrome is essential. While no autonomic symptoms were observed in this patient, the reported balance disturbances might suggest early cerebellar involvement. However, this remains speculative. Furthermore, no data are available on whether inflammatory cytokine levels were tested in this case, limiting the ability to draw direct correlations between MSA and the patient’s clinical presentation. Therefore, while it is less likely that this patient developed MSA, the possibility cannot be entirely excluded.

The hypothesis proposed in this case is based on the progressive and treatment-resistant nature of the motor syndrome, its overlap with HIV-related neurodegenerative conditions, and the extensive exclusion of other possible diagnoses. The combination of pyramidal signs, lack of response to levodopa, and imaging evidence of upper motor neuron involvement suggests a non-idiopathic etiology. Further research is required to define this phenotype and investigate the molecular mechanisms that may link chronic HIV infection to motor system degeneration.

## Conclusions

This case report describes an atypical motor syndrome in a patient with long-standing, well-controlled HIV infection, marked by both pyramidal and extrapyramidal signs and a poor response to dopaminergic therapy. The thorough exclusion of other common causes of parkinsonism and upper motor neuron syndromes supports the hypothesis that chronic HIV infection may contribute to neurodegeneration through mechanisms independent of active viral replication. While causality cannot be confirmed, the case highlights the importance of including HIV in the differential diagnosis of motor syndromes, particularly when clinical features are atypical or refractory to standard treatments. The absence of imaging findings typical of neurodegenerative disorders such as MSA, PSP, or corticobasal degeneration further underscores the need for heightened clinical awareness. Despite the limitation of being a single-patient report without confirmatory biomarkers or postmortem analysis, this case emphasizes the need for broader, longitudinal studies to better understand the neuropathological spectrum associated with HIV and to enhance early recognition and management of motor syndromes in patients receiving effective ART.
